# Response to: Comment on “First-Line *Helicobacter pylori* Eradication with Vonoprazan, Clarithromycin, and Metronidazole in Patients Allergic to Penicillin”

**DOI:** 10.1155/2018/8046838

**Published:** 2018-03-22

**Authors:** Soichiro Sue, Nobumi Suzuki, Wataru Shibata, Tomohiko Sasaki, Hiroaki Yamada, Hiroaki Kaneko, Toshihide Tamura, Tomohiro Ishii, Masaaki Kondo, Shin Maeda

**Affiliations:** ^1^Department of Gastroenterology, Yokohama City University Graduate School of Medicine, Yokohama, Japan; ^2^Department of Gastroenterology, Institute for Adult Disease, Asahi Life Foundation, Tokyo, Japan; ^3^Advanced Medical Research Center, Yokohama City University, Yokohama, Japan

We thank Dr. Kashani and Dr. Abadi [1] for their interest in our article [2]. In Japan, seven-day vonoprazan-containing triple therapy with clarithromycin and amoxicillin or vonoprazan-containing triple therapy with metronidazole and amoxicillin are approved regimens [3], but vonoprazan-containing triple therapy with clarithromycin and metronidazole is not approved and is used only in clinical trials which was approved by the ethics committee. Thus, people who cannot be given the standard triple therapy due to being allergic to penicillin are the subjects of this study. In addition, these patients cannot be covered by their medical insurance system for *Helicobacter pylori* eradication and related examinations, so it is difficult to add antibiotic susceptibility testing after the diagnosis of *H. pylori* infection for cost and ethical reason (this study was not designed to choose regimen by antibiotic resistance result). About the sample size, as we have also written the reason is the rate of patients allergic to penicillin, which makes it difficult to recruit many patients for this type of study. These are the explanations overall. We also answer each question:

- Generalization of the finding: We think the efficacy of this regimen is influenced by metronidazole resistance rates. In Japan, the metronidazole resistance rate is not high, and it is reported that metronidazole-containing triple therapy as a first-line therapy showed high eradication rate (ER) [4]. It is an important point that the vonoprazan, clarithromycin, and metronidazole regimen demonstrated excellent ER in areas with a high rate of clarithromycin resistance in two studies [2, 5], but these were performed in a country with a low rate of metronidazole resistance compared to other countries. We hope this regimen will be useful in countries with high clarithromycin but low metronidazole resistance rates as eradication regimen for those allergic to penicillin instead of PPI-based triple therapy with clarithromycin and metronidazole.
- Factors affecting the success of treatment: In all cases, patients did not drink alcohol during therapy, because drinking alcohol is not allowed with metronidazole under Japanese pharmaceutical rules. We did not report smoking status, but one of the twenty patients on the vonoprazan-based regimen was a smoker. The physician instructed them to stop smoking during therapy in this case based on past research [6]. We agree that stopping smoking during therapy is desirable.
- We reported the local (Kanagawa prefecture) clarithromycin resistance rate as 23.7% (95% CI: 18.5–29.7%, *n* = 236) in a previous article [3] and 40% in the Yokohama City University hospital where the study was performed [2]. We agree that a prospective study with clarithromycin and metronidazole resistance information is important, but it may be ethically difficult to use clarithromycin or metronidazole for drug-resistant *H. pylori*. In Japan, we have continued to use the VCM regimen for penicillin-allergic patients without testing susceptibility and continued to have a 100% successful rate without any severe adverse events.
- We agree that 95% CI of 86.1–100% result needs further confirmation at a large scale. But it is difficult to perform a large-scale study for patients allergic to penicillin.

We think it is important that two studies (ours on 20 patients and Ono et al. [5] on 14 patients) both showed high eradication rates. The studies cited in Table 1 are on vonoprazan-based regimens approved by Japanese insurance (except Ono et al.), and difficulty in recruiting the patients is very different between this study and the studies you cited due to the patient population. Actually, as you cited, we conducted a prospective study with 623 versus 608 patients of vonoprazan or PPI with clarithromycin and amoxicillin as first-line eradication and 180 versus 197 patients of vonoprazan or PPI with metronidazole and amoxicillin as cited in a meta-analysis [7], and we conducted an RCT with vonoprazan or PPI with clarithromycin and amoxicillin for clarithromycin-susceptible *H. pylori* with a total 106 of patients [8]. In addition, Ono et al. reported a total of 88 patients in their retrospective study, but the vonoprazan, clarithromycin, and metronidazole regimen only contained 14 patients.

(v) Diagnosis of *H. pylori* infection was performed based on Japanese guidelines (2016 ver). We think bacterial culture and drug susceptibility testing with agar dilution method from biopsy sample is optimal to assess the efficacy of the regimen as we did [8]. But as we described in this manuscript, it was ethically difficult to add the endoscopy and biopsy only for culture. The cases for which we performed culture and susceptibility testing were the patients who have not had endoscopy and who were not diagnosed as having *H. pylori* infection. In Japan, physicians can conduct *H. pylori* examination under national insurance coverage only after endoscopy. As shown in Table 1 of our research [2], all cases with endoscopy and diagnosis of *H. pylori* were confirmed by HpIgG, RUT, culture, pathology, UBT, urine antibody, or stool antigen. The reason we cannot conduct susceptibility testing in all cases was that endoscopy was already performed in another hospital in many cases (patients visited with referral letter). We did not perform endoscopic surgery as you wrote; we only performed biopsies for culture in some cases.

Taken together, this study shows the superiority of vonoprazan, clarithromycin, and metronidazole triple therapy compared to PPI-based therapy for patients who have an allergy to penicillin.
